# Host and pathogen genetic diversity shape vaccine-mediated protection to *Mycobacterium tuberculosis*

**DOI:** 10.3389/fimmu.2024.1427846

**Published:** 2024-06-28

**Authors:** Sara B. Cohen, Courtney R. Plumlee, Lindsay Engels, Dat Mai, Tara A. Murray, Ana N. Jahn, Bridget Alexander, Jared L. Delahaye, Lauren M. Cross, Karolina Maciag, Sam Schrader, Kaitlin Durga, Elizabeth S. Gold, Alan Aderem, Michael Y. Gerner, Benjamin H. Gern, Alan H. Diercks, Kevin B. Urdahl

**Affiliations:** ^1^ Seattle Children’s Research Institute, Center for Global Infectious Disease Research, Seattle, WA, United States; ^2^ Department of Medicine, Division of Infectious Diseases, University of Washington, Seattle, WA, United States; ^3^ Department of Pediatrics, University of Washington, Seattle, WA, United States; ^4^ Department of Immunology, University of Washington, Seattle, WA, United States

**Keywords:** tuberculosis, vaccine, heterogeneity, genetics, protection

## Abstract

To investigate how host and pathogen diversity govern immunity against *Mycobacterium tuberculosis* (Mtb), we performed a large-scale screen of vaccine-mediated protection against aerosol Mtb infection using three inbred mouse strains [C57BL/6 (B6), C3HeB/FeJ (C3H), Balb/c x 129/SvJ (C129F1)] and three Mtb strains (H37Rv, CDC1551, SA161) representing two lineages and distinct virulence properties. We compared three protective modalities, all of which involve inoculation with live mycobacteria: Bacillus Calmette-Guérin (BCG), the only approved TB vaccine, delivered either subcutaneously or intravenously, and concomitant Mtb infection (CoMtb), a model of pre-existing immunity in which a low-level Mtb infection is established in the cervical lymph node following intradermal inoculation. We examined lung bacterial burdens at early (Day 28) and late (Day 98) time points after aerosol Mtb challenge and histopathology at Day 98. We observed substantial heterogeneity in the reduction of bacterial load afforded by these modalities at Day 28 across the combinations and noted a strong positive correlation between bacterial burden in unvaccinated mice and the degree of protection afforded by vaccination. Although we observed variation in the degree of reduction in bacterial burdens across the nine mouse/bacterium strain combinations, virtually all protective modalities performed similarly for a given strain-strain combination. We also noted dramatic variation in histopathology changes driven by both host and bacterial genetic backgrounds. Vaccination improved pathology scores for all infections except CDC1551. However, the most dramatic impact of vaccination on lesion development occurred for the C3H-SA161 combination, where vaccination entirely abrogated the development of the large necrotic lesions that arise in unvaccinated mice. In conclusion, we find that substantial TB heterogeneity can be recapitulated by introducing variability in both host and bacterial genetics, resulting in changes in vaccine-mediated protection as measured both by bacterial burden as well as histopathology. These differences can be harnessed in future studies to identify immune correlates of vaccine efficacy.

## Introduction

Tuberculosis (TB), caused by the bacterium *Mycobacterium tuberculosis* (Mtb), remains a leading cause of morbidity and mortality across the globe (1), despite introduction of the Bacille Calmette-Guérin (BCG) vaccine over a century ago. BCG continues to be used in many countries due to its ability to curb disease, especially disseminated disease, in infants (2), yet it has unfortunately shown mixed efficacy at preventing pulmonary TB among adults, ranging from 0–80% (3–5). In the most commonly used mouse model of TB, C57BL/6 (B6) mice are aerosol-infected with ~50–100 colony-forming units (CFU) of lineage 4 Mtb (typically either H37Rv or Erdman). In this model, BCG immunization reliably reduces the pulmonary bacterial load by approximately 1 log at 4 weeks following infection, but this protection often wanes over time (6–8). However, the translational relevance of this pattern of protection remains unclear. Human TB disease often presents with a classic necrotizing granuloma, a feature that is notoriously absent in B6 mice, and yet pulmonary TB in humans is far more heterogeneous than often appreciated, with pathological features ranging from necrotic granulomas, non-necrotic granulomas, to pneumonia-like alveolitis (9). Despite known drawbacks of the mouse model (10), it remains an essential tool by providing opportunities for mechanistic study, and its relatively low cost enables larger scale experiments. To improve the model, we seek to better reflect the heterogeneity of human disease and provide a more robust platform for preclinical vaccine testing. To this end, we have introduced heterogeneity into the mouse model at multiple levels, including three host strains, three bacterial strains, and three protective modalities.

Host genetics are strong determinants of bacterial burden, pathology, and vaccine outcome. This has been highlighted by several studies (11–14), including a panel of subcutaneous (SC) BCG-vaccinated collaborative cross (CC) mice, where only half of the host strains showed significant reductions in lung CFU as a result of vaccination (15), and intravenous (IV) BCG-immunized diversity outbred (DO) mice, in which a wide range of CFU, survival outcomes, and pathology features was observed (16, 17). In our study, three mouse strains were included due to their broad range of responses to infection with Mtb. B6 mice can survive at least 1 year following aerosol challenge; this relative resistance to Mtb infection is thought to be at least partially due to inherent Th1 skewing and results in immunopathology characterized by diffuse lesions that lack the cellular organization of human granulomas (18). Despite this, the use of B6 mice offers an abundance of molecular tools, including transgenic and gene-knockout strains. C3HeB/FeJ (C3H) mice have high pulmonary bacterial loads and can form classical necrotizing granulomas with central caseation and hypoxia, as observed in advanced human TB disease, due at least in part to their high levels of type I interferons (19–21). The C129F1 line was generated by crossing Balb/c mice, which offer mechanistic tools, and 129/SvJ mice, which are relatively susceptible to Mtb compared to B6 mice, with poor survival outcomes (18, 22).

Mtb strain-specific properties also contribute to the heterogeneous outcomes of infection. First, different Mtb strains can elicit a range of distinct immune responses (23, 24). Second, different Mtb strains can predispose to varying patterns of cellular and tissue organization. For example, H37Rv infection of B6 mice results in disorganized lesions with intermixed macrophages and lymphocytes, while HN878 infection results in organized lesions with prominent B cell aggregates (25). Third, strains from different Mtb lineages can have varying impacts on vaccine-mediated immunity (26). We focused on lineages 2 and 4, which are considered the most pathogenic of all Mtb lineages (27). Lineage 4 (North American/European), the most geographically distributed lineage (28), is actually found on all continents, although the underlying mechanisms of its success as a human pathogen remain poorly understood. Lineage 2 (W-Beijing) strains are often associated with increased transmission and, accordingly, are growing in global distribution (29). Here, we incorporate bacterial heterogeneity by using three Mtb strains with divergent pathogenicity and immunogenicity: 1) H37Rv, a well-characterized lineage 4 strain that is commonly used for experimental studies; 2) CDC1551, a lineage 4 strain that is hyperimmunogenic but relatively low in virulence (30); and 3) SA161, a lineage 2 clinical isolate that is hypoimmunogenic and hypervirulent (31).

Finally, we examined three modes of protection (hereafter referred to as “vaccination” for simplicity). We immunized mice with BCG using two different routes: SC or IV. Despite the limitations of BCG and its varying efficacy in humans, more recent data have renewed interest in dissecting the mechanisms of protection it elicits in the limited settings where it is effective. For example, high-dose IV BCG was recently shown to confer superior protection to intradermal (ID) BCG in non-human primates (NHP) challenged with low-dose aerosolized Mtb (32), which correlated with increased airway-resident T cells and NK cells (33). Furthermore, BCG can prevent detectable infection in a small percentage of mice infected with an ultra-low dose (ULD) of aerosolized Mtb (8). Thus, understanding how and when BCG is effective could greatly advance our understanding of TB protective immunity. The third modality of protection included in our screen is the concomitant Mtb (CoMtb) model, in which a small (10^4^) inoculum of virulent H37Rv is administered ID to the ear (7). Inoculated bacteria are trafficked to the draining cervical lymph node where they persist for at least a year, without significant dissemination to the lung. Upon challenge with aerosolized Mtb, CoMtb mice display ~1–2 logs of protection that endures at least 4 months following infection. While not a true vaccine, this model is consistent with several observations that natural immunity induced by prior infection with Mtb itself can mediate protection against human TB disease. First, post-mortem studies observed that individuals with evidence of lymph node disease were less likely to have pulmonary TB (34). Second, nursing students who entered TB-endemic clinical settings with a positive tuberculin skin test (TST) were less likely to develop active TB disease than their classmates who were TST-negative, suggesting that pre-existing, subclinical infection with Mtb can provide protection against developing disease (35).

Altogether, this study examined 36 combinations of mouse strains, bacterial strains, and protective modalities, in which we assessed pulmonary bacterial burdens across time points (early, Day 28; and late, Day 98) and histopathology at Day 98. For most host/bacterial strain combinations, we found similar reductions in bacterial load across vaccine modalities. Histopathological analysis showed that vaccination variably impacted pathologic features, and bacterial burden at the early peak of infection was a better predictor of late pathological outcomes than bacterial burden measured at late time points. These results suggest that examination of vaccine responses in a single host/bacteria genetic background is likely insufficient to predict outcomes in heterogeneous human populations and highlight the importance of measuring both bacterial burdens and pathology when assessing the effectiveness of TB vaccines in animal models.

## Materials and methods

### Mice

C57BL/6J (B6, #000664) and C3HeB/FeJ (C3H, #000658) mice were purchased from Jackson Laboratories (Bar Harbor, ME). Balb/c x 129/SvJ (C129F1) mice were a custom breeding order from Jackson Laboratories (Bar Harbor, ME). Female mice between the ages of 9–12 weeks were used. All animals were housed and maintained in specific-pathogen-free conditions at Seattle Children’s Research Institute (SCRI). All animal studies were performed in compliance with the SCRI Animal Care and Use Committee.

### BCG immunizations

BCG-Pasteur was cultured in Middlebrook 7H9 with OADC supplement and 0.05% Tween-80 at 37°C with constant agitation for five days. BCG was back diluted in 7H9 for two days and grown to an OD of 0.2–0.5. Bacteria was diluted in PBS and mice were injected subcutaneously (SC) or intravenously (IV) with 200µl of 10^6^ CFU. After immunization, mice were rested for 8 weeks prior to Mtb infection.

### Concomitant Mtb model

The CoMtb model was established as described previously (7, 36). Briefly, mice were first anesthetized by intraperitoneal injection of ketamine and xylazine diluted in PBS. Mice were placed in a lateral recumbent position, and the ear pinna was flattened with forceps and pinned onto an elevated dissection board using a 22 G needle. H37Rv Mtb grown to an OD between 0.2–0.5 over a 48-hour period was diluted to 10^6^ CFU/ml in PBS, and 10 ul (10^4^ CFU) was administered into the dermis of the ear using a 26s G Hamilton syringe. Mice were then rested for 8 weeks prior to Mtb infection.

### Mtb aerosol infections

Infections were done with stocks of H37Rv, SA161 or CDC1551 as previously described (37). To perform aerosol Mtb infections, mice were placed in a Glas-Col aerosol infection chamber, and 50–100 CFU were deposited into their lungs. To confirm the infectious inoculum, two mice per infection were euthanized on the same day of infection, and their lungs were homogenized and plated onto 7H10 or 7H11 plates for determination of CFU.

### CFU plating

Right lungs were homogenized in M tubes (Miltenyi) containing 1mL PBS+0.05% Tween-80 (PBS-T) using a GentleMACS machine (Miltenyi). Homogenates were then diluted in PBS-T and plated onto 7H10 plates. Plates were incubated at 37°C for at least 21 days before quantification of CFU.

### Histology

Left lungs were fixed in 10% formalin for 24 hours, then dehydrated in 70% ethanol at 4°C for at least 24 hours. Samples were paraffin embedded and sectioned at the University of Washington Histology Core. Subsequently, slides were reviewed by a veterinary pathologist and scored in a blinded fashion based on the following metrics (see **Supplementary Table 2**): mixed granulomas (ill-formed granulomas with mixture of macrophages and lymphocytes), defined granulomas [well defined with increased separation of macrophages, epithelioid or multinucleated giant cells (MNGC) with lymphoid aggregates], perivascular lymphoid aggregates (PV LA), peribronchiolar lymphoid aggregates (PB LA), histiocytes, foamy macrophages, MNGC, alveolar hyperplasia, neutrophils, necrosis, cholesterol clefts, edema, extent 1 (percent involvement of the lung), extent 2 (percent involvement of the lung in the worst manner).

## Results

### Most vaccine modalities provide early protection against Mtb challenge, but the durability of protection varies according to host and bacterial strain

**Figure 1:**
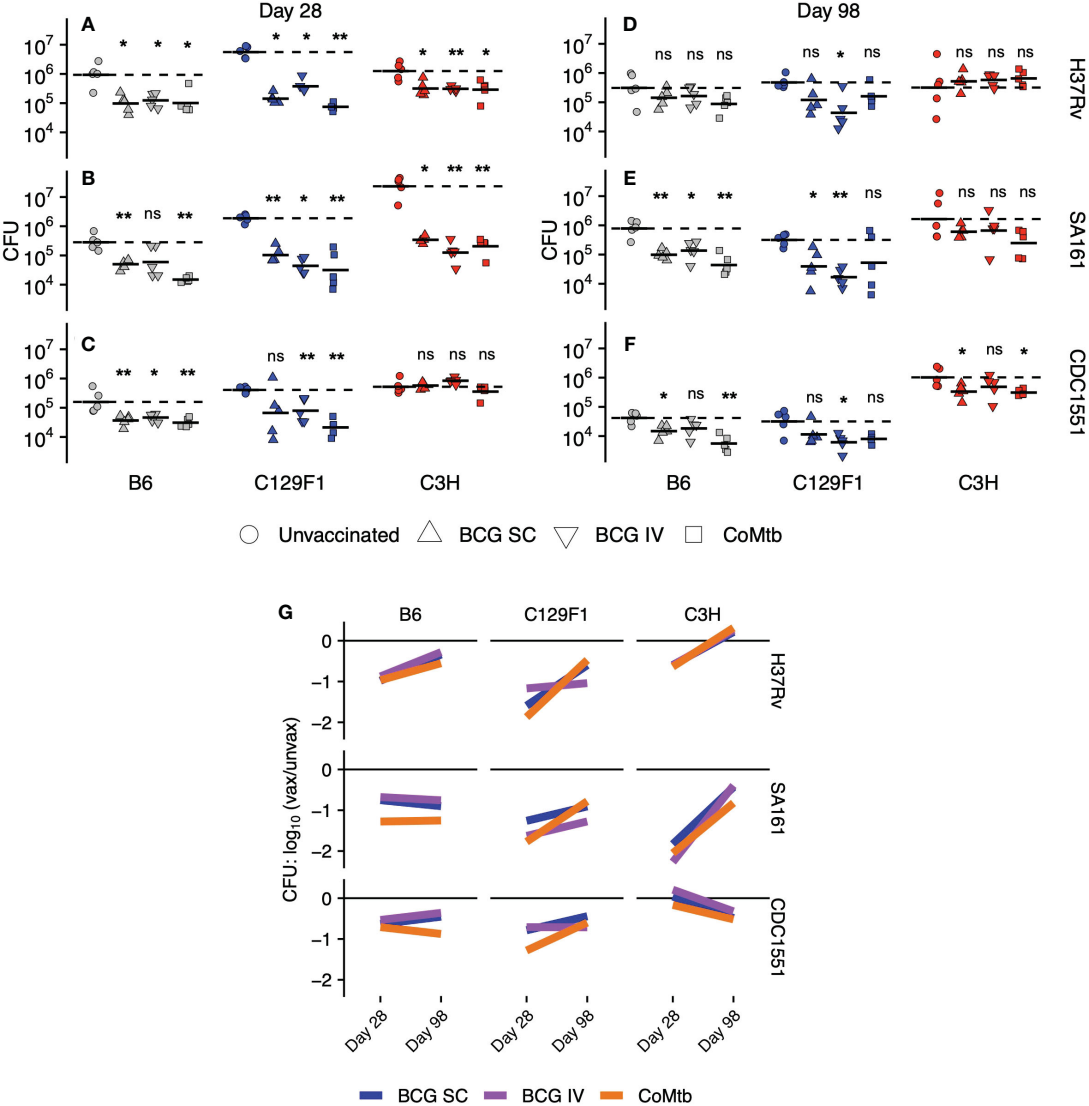
Lung bacterial burdens following vaccination across strain combinations. Right lungs were excised from C57BL/6 (B6), C3HeB/FeJ (C3H), and 129/SvJxBalb/c (C129F1) mice administered BCG [subcutaneous (SC) or intravenous (IV)] or intradermal concomitant Mtb (CoMtb) for 8 weeks and then aerosol-challenged with H37Rv, SA161, or CDC1551 Mtb for either **(A–C)** 28 days or **(D–F)** 98 days. Lungs were homogenized and plated for CFU enumeration. **(G)** Cumulative CFU data are plotted as a log change over unvaccinated for each host-bacterial strain combination over time. Statistical comparisons were performed by non-parametric Wilcoxon ranked test, with *, p<0.05; **, p<0.01; ns, not significant.

As an initial, quantitative measure of the degree and durability of protection afforded by each vaccine modality across the strain-strain combinations, we measured lung CFU burden at Day 28 and Day 98 following infection (**Figure 1**). Among unvaccinated mice at Day 28, we observed a wide range of outcomes, with bacterial loads differing by over 100-fold. For example, infection with H37Rv resulted in the highest CFU burden among C129F1 mice, (**Figure 1A**), while infection with SA161 yielded the highest CFU burden among C3H mice (**Figure 1B**); however, these two strains of mice responded with similar CFU burdens after infection with CDC1551 (**Figure 1C**). Across Mtb strains, B6 mice developed the lowest CFU burdens among the host genotypes at Day 28 (**Figures 1A–C**), while C3H mice infected with SA161 developed the highest overall lung burden across both time points (**Figures 1B, E**). There was much less heterogeneity in burdens among unvaccinated mice at Day 98, with equivalent loads across all genotypes in response to H37Rv (**Figure 1D**) and SA161 (**Figure 1E**). Infection with CDC1551 resulted in significantly higher lung bacterial loads in C3H mice than in the other mouse strains at the late (**Figure 1F**) but not early (**Figure 1C**) timepoint, suggesting that, even at late time points, we can identify differential responses to Mtb infection across combinations.

As expected from prior reports of vaccine-mediated immunity in the B6 mouse model (6–8, 38), lung bacterial burdens in B6 mice were significantly reduced at Day 28 by all vaccine modalities following challenge with all bacterial strains (**Figures 1A–C**), with the exception of IV BCG with SA161 challenge, where a high variability in CFU burden was seen (**Figure 1B**). The C129F1 mice behaved similarly to B6 mice based on CFU reductions following all vaccine modalities in response to all challenge strains. Unvaccinated C129F1 mice had particularly high bacterial loads, but these burdens were reduced to similar levels as B6 mice following vaccination (**Figures 1A–C**). In contrast to both B6 and C129F1 mice, C3H mice displayed a wide range of vaccine-mediated responses at Day 28 (**Figures 1A–C**). Unexpectedly, these mice were not protected from CDC1551 challenge by any vaccine modality at this time point (**Figure 1C**), whereas all modalities resulted in approximately 0.6-log CFU reduction upon H37Rv challenge (**Figure 1A**) and up to 2-log CFU reduction upon SA161 challenge (**Figure 1B**) (**Supplementary Table 1**). Overall, at Day 28, 22/27 (81.4%) strain-strain combinations resulted in significant decreases in lung CFU. At Day 98, however, CFU reduction in response to vaccination was only seen in 11/27 (40.7%) combinations. Some strain-strain combinations failed to display any vaccine-mediated reduction in bacterial load at Day 98, such as B6 and C3H mice infected with H37Rv (**Figure 1D**) and C3H mice infected with SA161 (**Figure 1E**). Among strain combinations that were protected at Day 98, there was heterogeneity associated with vaccine modality. For example, IV BCG mediated substantial protection in C129F1 mice infected with H37Rv (**Figure 1D**) and CDC1551 (**Figure 1F**), whereas SC BCG and CoMtb did not (**Figures 1D, F**).

We next plotted lung CFU in vaccinated vs unvaccinated mice within each strain-strain combination at each time point to visualize trends in the long-term durability of vaccine-mediated protection (**Figure 1G**). Among all strain-strain combinations, the magnitude of protection generally decreased over time [6/9 (B6-H37Rv, C129F1-H37Rv, C3H-H37Rv, C129F1-SA161, C3H-SA161, C129F1-CDC1551), 67%]; however, in some cases it stayed constant [2/9 (B6-SA161, B6-CDC1551), 22%] or even increased [1/9 (C3H-CDC1551), 11%]. While we noted heterogeneity in vaccine-mediated outcomes across strain-strain combinations and time points, as described in **Figures 1A–F**, there was a striking overlap in the behavior of the three protective modalities within most combinations. Thus, while bacterial and host strain diversity had a large overall impact on vaccine-mediated outcomes, the vaccines themselves contributed relatively little to the observed heterogeneity. For example, we observed similar initial log reductions in CFU followed by waning protection over time, as visualized by a positive slope, for B6 mice infected with H37Rv for all 3 modalities. Surprisingly, in the C3H/SA161 group, which was the most strongly protected combination at Day 28, none of the vaccines were able to mediate protective efficacy at the later time point. In contrast, durable protection across vaccine modalities, as visualized by a slope of ~0, was only seen in the B6/SA161 and B6/CDC1551 combinations. We found this was also true for almost all vaccines in C129F1 and C3H mice challenged with H37Rv (**Figure 1G**). The only example of improved protection over time, as indicated by a negative slope, was C3H mice challenged with CDC1551, although this combination failed to show evidence of vaccine efficacy at the early time point.

**Figure 2:**
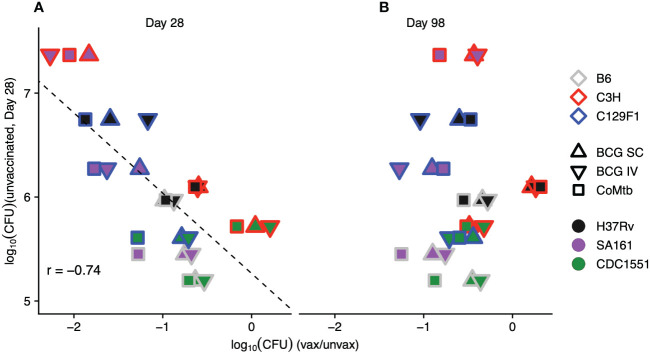
The magnitude of vaccine-induced reduction in bacterial load correlates with initial burden in unvaccinated mice. The average log reduction in CFU for each mouse strain/protective modality/bacterial strain combination at Day 28 **(A)** and Day 98 **(B)** is plotted against the bacterial burden for each mouse strain/bacterial strain combination in unvaccinated mice at Day 28. Each point represents the average of 5 mice/condition. Outline colors represent mouse strain, fill colors represent bacterial strain, and shapes represent protective modality.

As previously noted, unvaccinated C3H mice infected with SA161 developed the highest bacterial burdens of any strain-strain combination at Day 28, peaking at loads approximately 1-log higher than in either B6 or C129F1 mice (**Figure 1B**). Furthermore, this combination demonstrated the strongest vaccine-mediated reduction in lung CFU at Day 28 of ~2 logs (**Supplementary Table 1**). To address whether early bacterial load in unvaccinated mice correlated with vaccine-mediated CFU reduction across strain-strain combinations, we plotted the log fold-change of CFU upon vaccination against the lung CFU of unvaccinated mice (**Figure 2A**). This analysis confirmed a correlation (r=-0.74) between the initial bacterial load at Day 28 in unvaccinated mice and vaccine-induced reduction in bacterial load. This correlation was not observed at the late, Day 98 time point (**Figure 2B**), suggesting that the protective mechanisms induced by vaccination may be more effective at curbing rapid bacterial growth early during infection than in reducing bacterial burden in an established infection.

**Figure 3:**
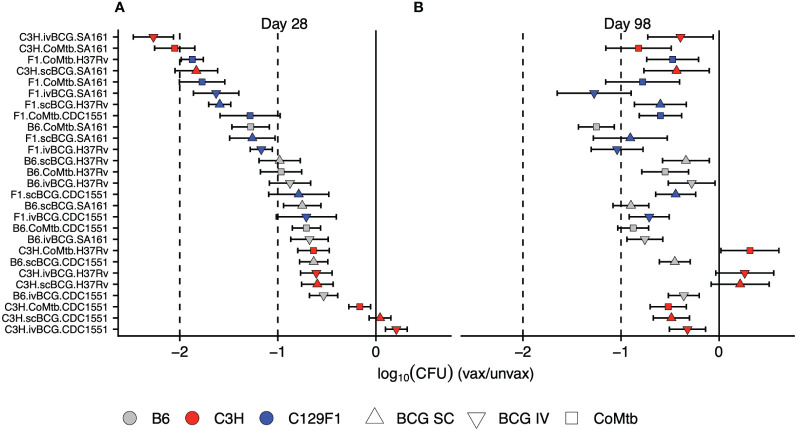
Early vaccine-mediated reductions in bacterial load do not predict late outcomes. The strain-strain combinations were ranked in order of highest vaccine-mediated reduction in bacterial load at **(A)** Day 28 post-infection. Keeping the same order, these combinations were then plotted to include the Day 98 log reductions in bacterial burden **(B)**.

We next ranked the protective efficacy of each vaccine modality in each strain-strain combination in order of its lung CFU burden in vaccinated vs unvaccinated mice at Day 28 (**Figure 3A**). These data underscore the broad range of magnitudes of vaccine-mediated protection at early time points across strain-strain combinations, with bacterial load reductions ranging from 0 to >2 logs, highlighting the critical role of host and pathogen diversity in modulating vaccine outcomes (**Figure 3A**). In contrast, the combinations at Day 98 were largely restricted to between 0 and <1 log of protection, highlighting both the limited range of responses and overall waning of protection at later time points (**Figure 3B**). Importantly, the disordered nature of vaccine efficacy at Day 98 (**Figure 3B**) when ranked by vaccine efficacy at Day 28 (**Figure 3A**) illustrates the inability of early protective responses to predict late vaccine-mediated protection. This heterogeneity in vaccine outcomes that emerges upon incorporating host and genetic diversity underscores the limitations of traditional animal model vaccine testing that solely relies on the standard B6/H37Rv strain combination.

### Pathological changes are driven by host and bacterial diversity

**Figure 4:**
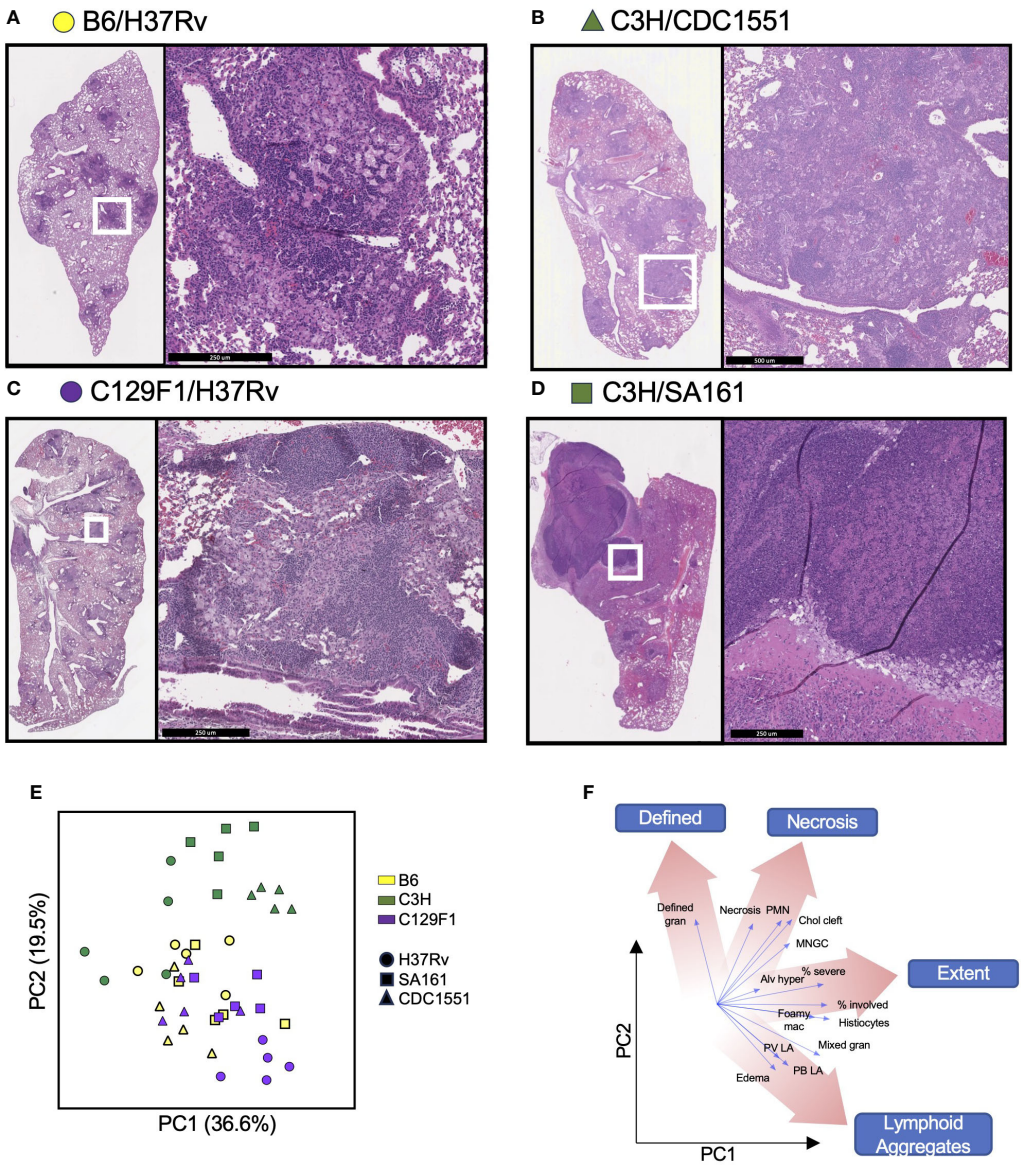
Mouse strains present a range of histopathological features upon infection with different Mtb strains. The left lung of mice was preserved in formalin at Day 98 post-Mtb infection and subjected to hematoxylin and eosin (H&E) staining to observe 14 histopathological features, which were graded in a blinded fashion by a licensed pathologist. **(A–D)** Representative sections from each strain of mouse is shown to illustrate the varying histological presentations. **(E)** Cumulative scores were used to generate a principal component analysis (PCA) encompassing all unvaccinated mice infected with each Mtb strain, with **(F)** different regions representing a predominance of certain pathological features.

At the Day 98 time point, a time at which lesions have fully developed, the left lung was preserved in formalin for hematoxylin and eosin (H&E) staining and histopathologic analysis. The following features were assessed: extent, mixed granulomas, defined granulomas, perivascular lymphoid aggregates, peribronchial lymphoid aggregates, histiocytes, multinucleated giant cells (MNGC), neutrophils, alveolar hyperplasia, necrosis, cholesterol clefts, and edema (**Supplementary Table 2**). Representative H&E images from unvaccinated mice that embody clear histological patterns are shown in **Figure 4**. B6 mice infected with H37Rv had moderate involvement with disorganized lesions (**Figure 4A**), C3H mice infected with CDC1551 had extensive involvement with MNGC (**Figure 4B**), and C129F1 mice infected with H37Rv had a high degree of lung involvement driven by lymphoid aggregates (**Figure 4C**). Consistent with reports that C3H mice develop caseating granulomas more typical of primary TB granulomas seen in humans (19, 21, 39), C3H mice infected with SA161 displayed distinctive granuloma structures defined by overwhelming necrosis and neutrophil infiltration (**Figure 4D**). This strain combination reached the highest bacterial burdens at the Day 28 time point, nearly 1 log higher than all other combinations (**Figure 1B**), suggesting that early bacterial burdens can drive pathology at later time points. To gain insight into the pathological features driving lesion structure in each genotype, we performed a principal component analysis (PCA) using all scored pathology variables for all mice (**Supplementary Table 2**), first showing unvaccinated mice for simplicity (**Figure 4E**). By examining the PCA loading scores, we found that individual pathological features tended to group into 4 distinct categories: Defined, Necrosis, Extent, and Lymphoid aggregates (**Figure 4F**). For unvaccinated mice, we found that variation in PCA scores was largely driven by mouse strain, and to a lesser extent by bacterial strain (**Figure 4E**). Overall, C3H mice clustered separately from B6 and C129F1 mice, typified by a high extent of involvement and the presence of neutrophils (**Figures 4B, E, F**). Conversely, C129F1 mice contained lymphocyte-rich lesions with prominent peribronchial and perivascular aggregates (**Figures 4C, E, F**). Lastly, B6 mice largely fell near the center of the PCA plot, consistent with the lack of prominent scored features and the presence of disorganized lesions, with histiocytes intermixed with loose lymphoid regions (**Figures 4A, E, F**).

### The ability of vaccine modalities to improve pathology outcomes is dependent on both host and bacterial strain

**Figure 5:**
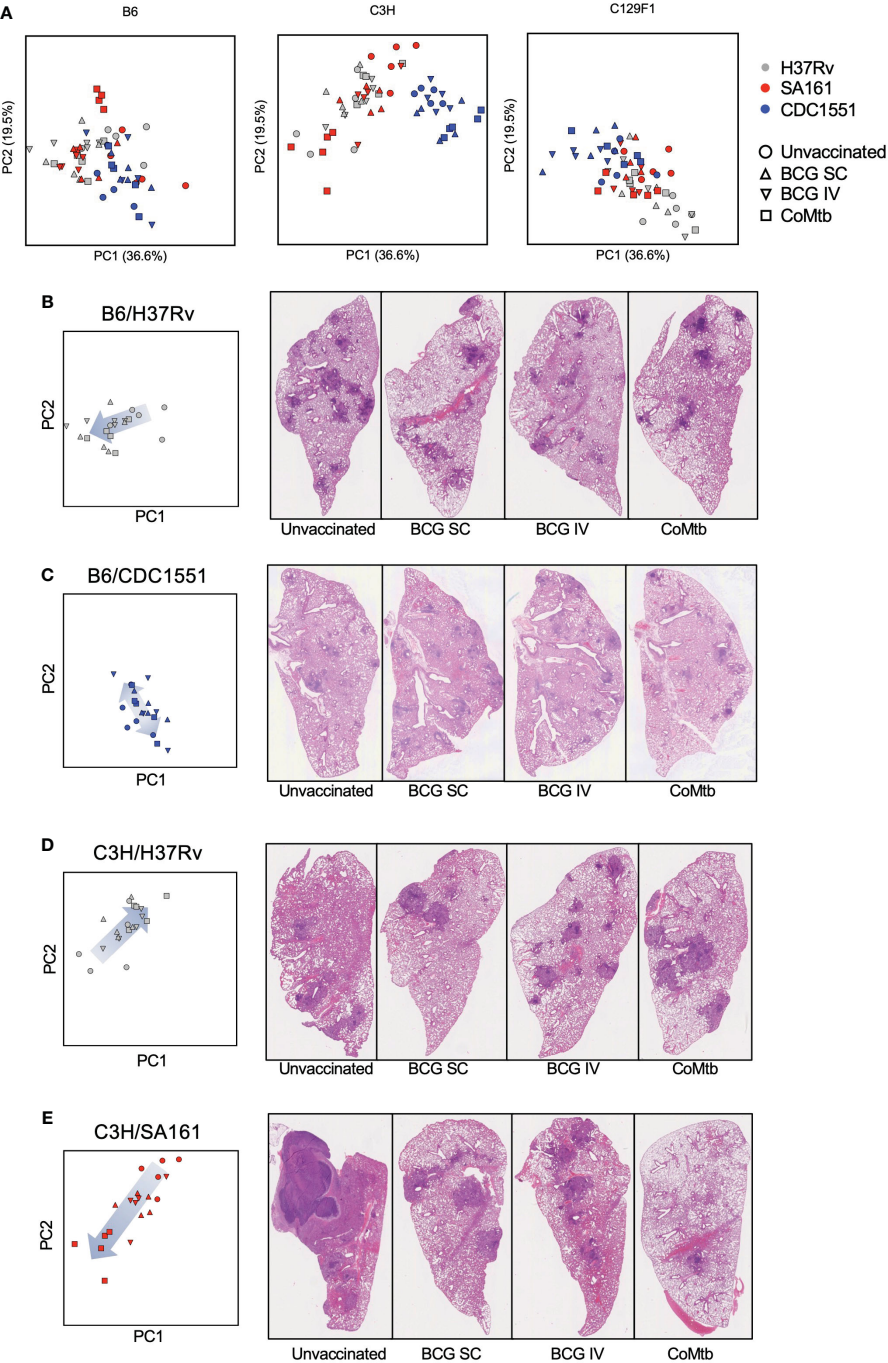
Distinct strain-strain combinations result in unique vaccine-mediated histological changes. **(A)** The same PCA plot shown in **Figure 4** is now displayed separately for each mouse strain and includes vaccinated cohorts, with color depicting bacterial strain and shape depicting protective modality. **(B–E)** Shown are representative Day 98 H&E images for select strain-strain combinations of interest, alongside their associated PCA plots. The arrows indicate the change in direction of samples within the PCA plot following vaccination.

To determine how the different vaccine modalities impacted pathology outcomes, we included data points from vaccinated mice onto the PCA plot, and separated them for each mouse strain to facilitate visualization (**Figure 5A**). For B6 mice, there was large overlap in pathology outcomes regardless of bacterial strain or vaccination regimen. In C3H mice, CDC1551 infection led to pathology scores that clustered in a region of the plot associated with higher lesion extent, regardless of vaccine strategy, while both H37Rv and SA161 infection resulted in a wide range in pathology scores depending on vaccination modality. In C129F1 mice, Mtb strain, but not vaccination modality, was the most significant driver of outcome along these principal components (**Figure 5A**). To gain insight into the vaccine-mediated changes in pathology in individual strain-strain combinations, we examined the relevant subset of data points from the PCA plot alongside representative H&E images from Day 98 (**Figures 5B–E**; **Supplementary Figure 1**; **Supplementary Table 2**). Several of the most striking combinations are shown in **Figure 5**. For the B6/H37Rv combination, all vaccine modalities resulted in modest yet clear changes in pathology scores, largely driven by decreased extent of lung involvement and lesion definition, with the arrow indicating a shift from greater to lesser involvement with vaccination (**Figure 5B**). For B6 mice infected with CDC1551, histologic features were similar regardless of vaccination and were marked by poorly defined lesions and abundant myeloid aggregates (**Figure 5C**). In the C3H/H37Rv combination, there was an unexpected increase in involvement and degree of defined granulomas in vaccinated vs unvaccinated mice (**Figure 5D**). This aligns with the complete lack of protection from bacterial burden in this combination at Day 98, and even a trend toward increased CFU upon vaccination (**Figure 1D**). Finally, in the C3H/SA161 combination, there was dramatic overall improvement in pathology resulting from all vaccine modalities; the large necrotic granulomas observed in the unvaccinated group were absent in vaccinated mice (**Figure 5E**). CoMtb-inoculated C3H mice infected with SA161 had the largest reversal in degree of lesion extent, scoring lower than either BCG-immunized cohort (**Figure 5E**). The remaining strain-strain combinations and their respective PCA plots and H&E images are shown in **Supplementary Figure 1**. Together, this shows that host and bacterial genetics play a significant role in determining the effect of vaccines on pathology, and that while vaccines appeared to improve pathology in most cases, they had no effect and potentially exacerbated pathology in certain strain-strain combinations.

### Correlations of pathology features and bacterial burden vary by host strain and time point

**Figure 6:**
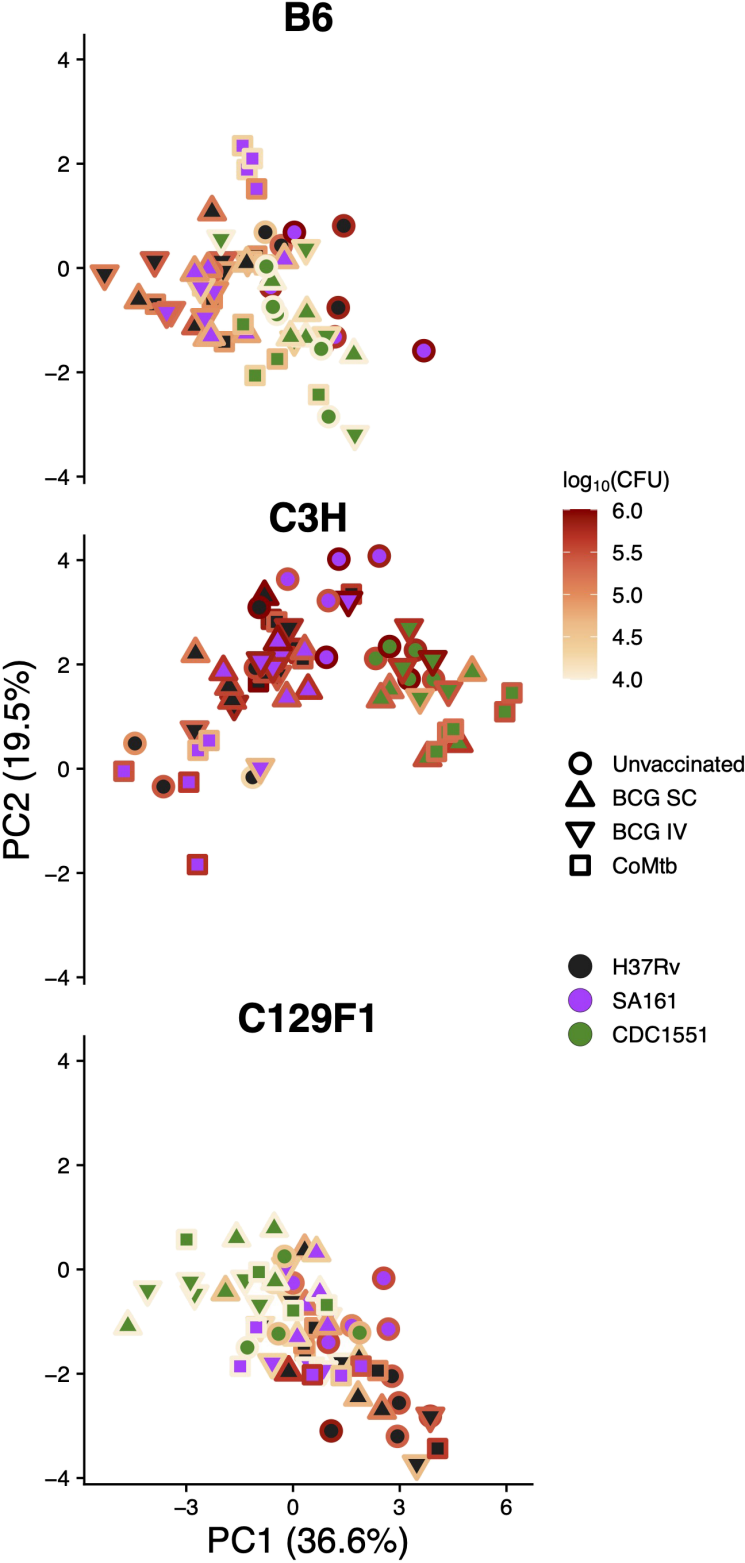
Pathology shows minimal correlation with bacterial burden late during infection. The PCA plots of histology scores separated by mouse genotype, as shown in **Figure 5**, were placed alongside the same plots now overlaid with the matched lung CFU value for each sample from Day 98, with dark colors representing lower bacterial CFU and bright colors representing higher bacterial CFU.

Given the diverse histologic outcomes noted across strain-strain combinations, we sought to determine whether any of these patterns directly associated with lung bacterial burden outcomes. To address this, we overlaid a heatmap of lung CFU values at Day 98 for each mouse genotype onto their respective subset of the histopathology score PCA plot (**Figure 6**). In B6 mice, there was an overall lack of correlation between bacterial burden and pathology features. In contrast, while high bacterial burdens in unvaccinated C3H/SA161 mice associated with necrosis, vaccination in this strain-strain combination led to both a decrease in CFU and a shift away from necrosis. No correlation was observed between histopathology PCA scores and either CFU or vaccination status for C3H/H37Rv mice, which otherwise overlapped with C3H/SA161 mice on the PCA plot. C3H/CDC1551 infection resulted in histopathology marked by high extent regardless of vaccination status. In C129F1 mice, infection with H37Rv was associated with both higher CFU and histopathology dominated by lymphoid aggregates, regardless of vaccination status or modality.

**Figure 7:**
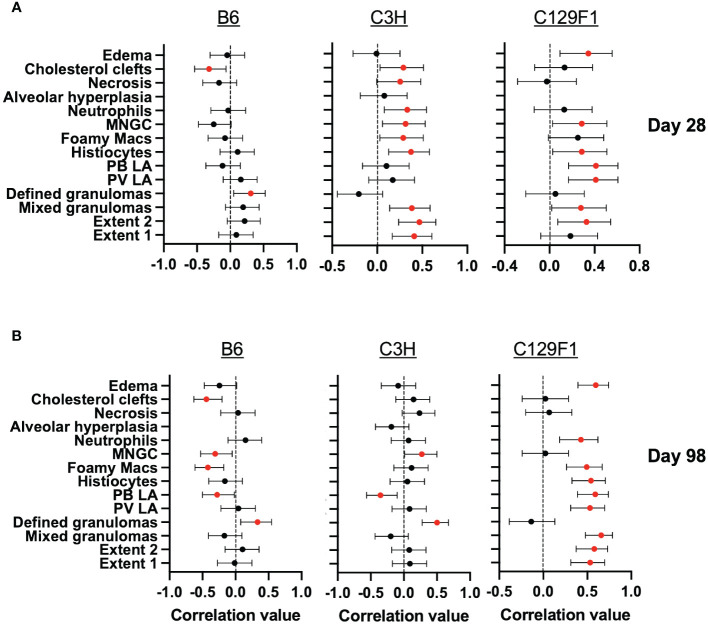
Late pathological features correlate more strongly with early than late bacterial burdens. Spearman correlation analysis was performed between histology scores for each pathology feature and lung bacterial burdens at **(A)** Day 28 and **(B)** Day 98 for each mouse genotype. For Day 98, the pathology scores and CFU values are paired, whereas for Day 28, we used the geometric mean of CFU from 5 mice per group to generate correlation values, since we did not have matched CFU values at this time point. Statistically significant correlations are shown in red (p<0.05).

To more clearly identify the histopathologic features that correlate with CFU outcome among mouse strains across Mtb genotypes and vaccine modalities, we performed Spearman correlation analysis of each scored feature with either the mean CFU value for all mice with that genotype at Day 28 (because matching H&E images were not collected at Day 28) (**Figure 7A**) or the paired CFU at Day 98 (where individual CFU values had a matching histopathology score) (**Figure 7B**). Consistent with the CFU heatmap overlay in **Figure 6**, B6 histopathology scores did not correlate with CFU at either time point, suggesting that the histological presentation of these mice is a poor indicator of bacterial control. Interestingly, in C3H mice, Day 28 CFU significantly correlated with several histological features, including necrosis, neutrophils, defined granulomas, and extent; however, these features did not correlate with Day 98 CFU burden. In C129F1 mice, on the other hand, most histopathology features correlated positively with CFU burden at both early and late time points, consistent with the observation that these mice retained a higher level of vaccine-induced protection at Day 98 (**Figures 1D–F**). Some correlative features were shared between C3H and C129F1 mice at Day 28, including mixed granulomas, MNGC, histiocytes, and extent 2, but certain features were uniquely correlated with bacterial load in each genotype. For example, burden in C3H mice uniquely correlated with neutrophils and necrosis, whereas burden in C129F1 uniquely correlated with both perivascular and peribronchial lymphocyte aggregates. Taken together, these data suggest that host genotypes display distinct pathological signatures, and high CFU burden can be associated with a range of unique pathologic indicators.

## Discussion

The mouse remains the main small animal model for immunological research due to the extensive availability of molecular reagents, a large body of historical data, and relatively low cost. Much of our current understanding of TB immunology had its origins in the mouse model before subsequent validation in humans, i.e., the critical roles for CD4 T cells (40), IFNγ (41, 42), IL-12 (43), and TNFα (44). The mouse is also used extensively to test and mechanistically dissect vaccine-mediated protection against TB disease. The standard dose (SD) mouse model of Mtb infection, in which animals are infected with ~50–100 CFU, yields a highly reproducible course of disease that enables robust experimental designs using modest numbers of mice. However, the current mouse model for measuring vaccine-mediated immunity, like most experimental animal models, does not account for the extensive host and pathogen variation that is seen in humans. Another problem is that vaccine-induced reductions in bacterial burdens in mice infected with 50–100 CFU are modest (~1–2 logs) and usually transient, disappearing at later time points. In this study, we show that host and Mtb strains are more important in this model than the vaccines themselves, or their routes of delivery, in dictating outcomes. This contrasts with studies in the NHP model (32), where BCG-mediated protection was significantly enhanced upon intravenous BCG administration relative to subcutaneous. This raises questions about whether the mouse model truly is able to measure aspects of vaccine-induced immunity that are relevant to protection against human TB and may help explain why current mouse models have performed poorly in predicting vaccine efficacy outcomes in human trials. By identifying shortcomings of the mouse model, this study also provides opportunities to make adjustments for potential improvements. For example, future studies should test whether BCG given intravenously at higher doses might improve outcomes and better reflect findings in NHP. It is also possible that differences would emerge in IV BCG-immunized mice after challenge with a more physiologic Mtb challenge dose (8).

Across the 27 mouse strain/Mtb strain/vaccine combinations, the protective effect of the modalities tested, as measured by CFU burden at Day 28, ranged from over 100-fold to nil. However, protection at Day 28 did not correlate with bacterial control at Day 98, suggesting that assessing late time points in this model may be important. However, Day 98 measurements across all vaccinated conditions only reached approximately 0.5-log CFU reductions, suggesting a limit to the achievable long-term protection in these models, and even this degree of reduction might potentially decrease further if even later time points were examined. Taken together with our observation that there were only a few instances of long-term protection and that these rarely exceeded 0.5-log CFU reduction at Day 98, the protective responses induced by BCG or CoMtb may be most effective at restraining rapidly growing bacteria during the first few weeks after infection. Indeed, across the mouse strain/Mtb strain combinations tested, the magnitude of vaccine-mediated protection at Day 28 was inversely correlated with the bacterial burden in unvaccinated mice, independent of protective modality. This is consistent with a previous finding that Mtb strains with the most robust growth *in vivo* at Day 28 were also the most restricted by BCG vaccination (45). While one interpretation of these findings is that the mouse model fails to reveal cases of remarkable vaccine-mediated immunity, one could also argue that some aspects of vaccine-induced immunity are masked by the use of a supraphysiologic Mtb challenge dose that could overwhelm the immune system. Some studies suggest that using a more physiologic challenge dose [ultra-low dose (ULD) infection with 1–3 CFU] may be able to discriminate differences in vaccine-induced immunity better than a 50–100 CFU challenge dose (8, 46). Thus, this study raises two important questions that should be addressed in future studies: 1) do mechanisms of vaccine-mediated immunity operate differently in settings of SD vs ULD? and 2) does a vaccine’s ability to restrict rapid bacterial growth early during infection predict its ability to prevent disease in humans?

Our study suggests that assessing vaccine-induced histopathologic changes may be helpful in evaluating vaccine efficacy as we observed a strikingly broad range of pathologic outcomes. These ranged from a dramatic decrease in pathology due to vaccination in the C3H/SA161 combination, minimal to no changes in the majority of combinations, to even apparent exacerbation of pathology in the C3H/H37Rv combination. It was surprising that pathology outcomes at Day 98 correlated poorly with bacterial burdens at this late time point, and in fact, were more strongly associated with bacterial burdens at Day 28. This suggests that host-pathogen interactions taking place at early time points may shape developing lesions and drive pathologic differences that are perpetuated at late time points. However, in B6 mice, overall lung involvement, as measured by Extent 1 scores, did not correlate with CFU at Day 28, which was unexpected given the expectation that both of these readouts indicate poor outcomes. This may reveal a caveat of using Day 98 tissue sections to correlate with Day 28 CFU, and future studies should use paired pathology scores for each time point. Further work is needed to understand the mechanistic underpinnings of these observations, which suggest that differences in host and bacterial genetics might shape the timing, type, and/or magnitude of immune responses modulated by pre-existing immunity or vaccination, which in turn can significantly affect pathologic outcomes. Given the availability of immunologic and genetic tools available for these mouse strains, this potentially allows for the evaluation of host-pathogen interactions within various microenvironments contained in these different structures, and for the mechanistic dissection of factors driving these differences. Furthermore, our work gives insight into combinations with the greatest potential to evaluate specific pathologic features of interest, for example, the C3H/SA161 combination to evaluate lesion necrosis, and C129F1/H37Rv for lymphoid aggregates. Together with the aforementioned studies in DO and CC mice, these results suggest that the long-maligned lack of diverse structures generated during Mtb infection in the mouse model is due to a focus on models that lack genetic heterogeneity. Together, these results suggest it is important to evaluate vaccine candidates with a diverse set of genetic backgrounds, and that success or failure in one strain of mouse infected with one strain of Mtb might not be predictive of clinical success or failure (47, 48). Furthermore, while bacterial burden is the most commonly used surrogate for protection in mice, it is equally important to uncover the presence of inflammatory features and destructive tissue pathology that may provide anatomic reasons for exacerbated disease, difficulty of treatment, and/or lasting sequelae even after the infection is resolved.

Overall, our study demonstrates that host and pathogen genetic differences play a large role in dictating outcomes after vaccination. The field must continue to develop Mtb infection models that reflect this heterogeneity. However, the fact that different protective modalities performed very similarly with each host-Mtb strain combination suggests that meaningful aspects of vaccine-induced immunity may be masked in mice infected with 50–100 CFU. A physiologic challenge dose may facilitate the assessments of more clinically relevant parameters of infection, such as prevention of dissemination and prevention of detectable infection, as well as induce more discrete histopathologic features (49). Thus, future studies are needed to compare the performance of vaccines to SD vs ULD challenge and to assess whether distinct mechanisms of protection can only be assessed in response to certain doses. One limitation of this study was the inclusion of only female mice. Due to the extensive scale of the study and the already inherent variability of host and bacterial components, it was not feasible to expand the groups to incorporate both sexes. We also limited our focus to CFU and histology as the two measured parameters of protection, and additional studies will be needed to examine immune correlates relating to these findings. Performing vaccine testing using several host and Mtb strain combinations in both sexes and doing so with a more physiologic Mtb challenge dose would certainly add complexity and cost to preclinical vaccine testing in mice. However, if future studies reveal that this approach facilitates an improved ability to discriminate protective differences between vaccines and results in better correlates of outcomes in human vaccine trials, adding such complexity may be essential.

## Data availability statement

The original contributions presented in the study are included in the article/**Supplementary Material**. Further inquiries can be directed to the corresponding authors.

## Ethics statement

The animal study was approved by Seattle Children’s Institutional Animal Care and Use Committee (IACUC). The study was conducted in accordance with the local legislation and institutional requirements.

## Author contributions

SC: Conceptualization, Formal analysis, Investigation, Writing – original draft, Writing – review & editing. CP: Conceptualization, Formal analysis, Investigation, Writing – original draft, Writing – review & editing. LE: Investigation, Writing – review & editing. DM: Investigation, Writing – review & editing. TM: Investigation, Writing – review & editing. AJ: Investigation, Writing – review & editing. BA: Investigation, Writing – review & editing. JD: Investigation, Writing – review & editing. LC: Investigation, Writing – review & editing. KM: Writing – review & editing. SS: Investigation, Writing – review & editing. KD: Investigation, Writing – review & editing. EG: Conceptualization, Writing – review & editing. AA: Funding acquisition, Writing – review & editing. MG: Conceptualization, Writing – review & editing. BG: Conceptualization, Formal analysis, Investigation, Writing – original draft, Writing – review & editing. AD: Conceptualization, Formal analysis, Supervision, Writing – original draft, Writing – review & editing. KU: Conceptualization, Funding acquisition, Supervision, Writing – original draft, Writing – review & editing.

## Supplementary material

Supplementary Figure 1 Representative H&E images of the remaining strain-strain combinations. H&E images of Day 98 lungs from the remaining combinations of mouse and bacterial strain are shown alongside their respective PCA plots.
