# Supplementary figures and images for: Host and pathogen genetic diversity shape vaccine-mediated protection to Mycobacterium tuberculosis
Source: Front Immunol. 2024 Jun 28;15:1427846. doi: 10.3389/fimmu.2024.1427846 (PMC11239334; doi:10.3389/fimmu.2024.1427846)

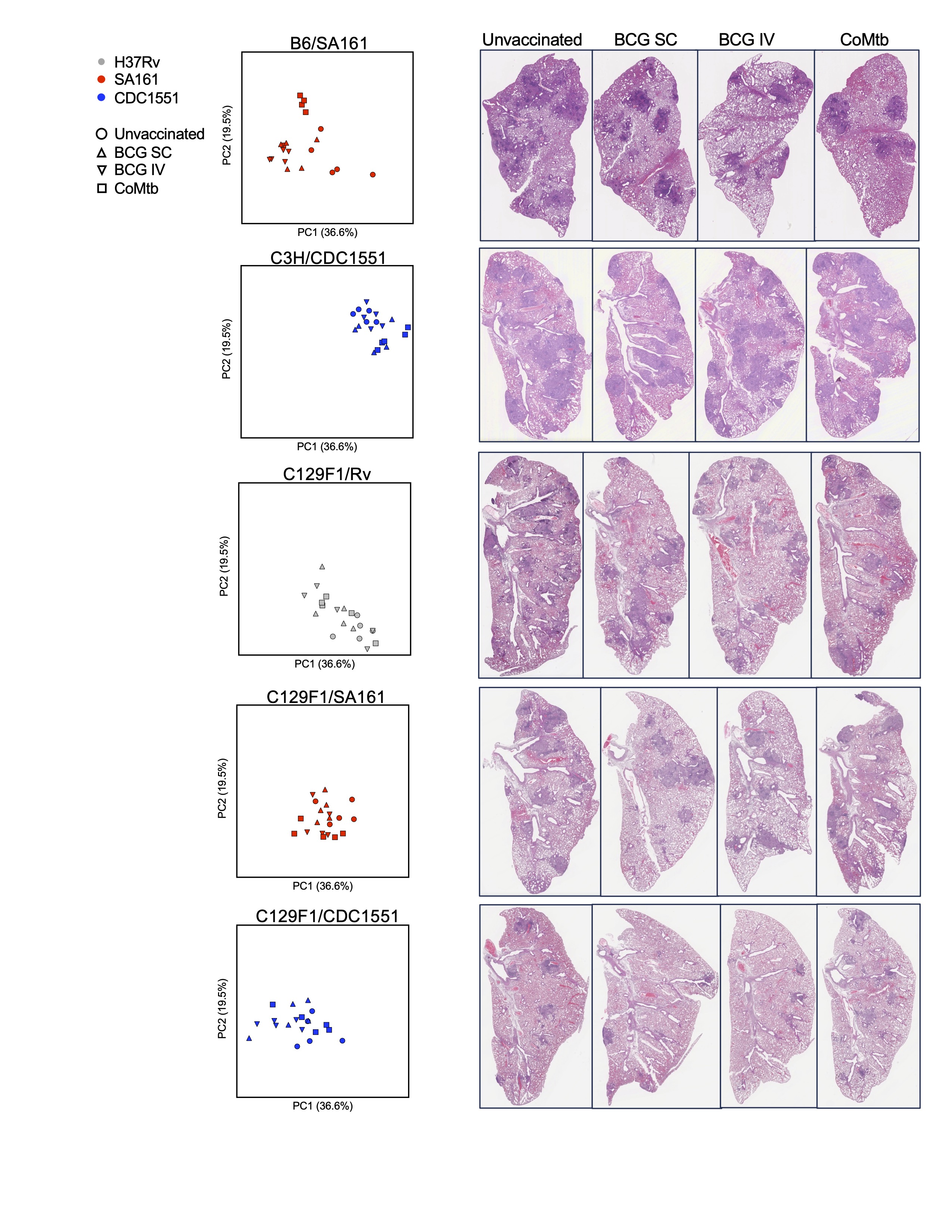

Supplement: Supplementary Figure 1 — Representative H&E images of the remaining strain-strain combinations. H&E images of Day 98 lungs from the remaining combinations of mouse and bacterial strain are shown alongside their respective PCA plots. [file Image_1.jpeg]
